# Detection of motor imagery based on short-term entropy of time–frequency representations

**DOI:** 10.1186/s12938-023-01102-1

**Published:** 2023-05-04

**Authors:** Luka Batistić, Jonatan Lerga, Isidora Stanković

**Affiliations:** 1 University of Rijeka - Faculty of Engineering, Vukovarska 58, 51000 Rijeka, Croatia; 2 Center for Artificial Intelligence and Cybersecurity, University of Rijeka, R. Matejčić 2, 51000 Rijeka, Croatia; 3 University of Montenegro, Džordža Vašingtona bb, 81000 Podgorica, Montenegro

**Keywords:** Brain–computer interface, Electroencephalography, Information entropy, Motor imagery, Movement detection, Time–frequency representations

## Abstract

**Background:**

Motor imagery is a cognitive process of imagining a performance of a motor task without employing the actual movement of muscles. It is often used in rehabilitation and utilized in assistive technologies to control a brain–computer interface (BCI). This paper provides a comparison of different time–frequency representations (TFR) and their Rényi and Shannon entropies for sensorimotor rhythm (SMR) based motor imagery control signals in electroencephalographic (EEG) data. The motor imagery task was guided by visual guidance, visual and vibrotactile (somatosensory) guidance or visual cue only.

**Results:**

When using TFR-based entropy features as an input for classification of different interaction intentions, higher accuracies were achieved (up to 99.87%) in comparison to regular time-series amplitude features (for which accuracy was up to 85.91%), which is an increase when compared to existing methods. In particular, the highest accuracy was achieved for the classification of the motor imagery versus the baseline (rest state) when using Shannon entropy with Reassigned Pseudo Wigner–Ville time–frequency representation.

**Conclusions:**

Our findings suggest that the quantity of useful classifiable motor imagery information (entropy output) changes during the period of motor imagery in comparison to baseline period; as a result, there is an increase in the accuracy and F1 score of classification when using entropy features in comparison to the accuracy and the F1 of classification when using amplitude features, hence, it is manifested as an improvement of the ability to detect motor imagery.

## Background

Injuries and disorders that impair the use of motor functions can significantly alter the lives of individuals affected. Therefore, many research groups are trying to tackle the problem of restoring or replacing the lost functionality using different methods. One of such methods is a brain–computer interface (BCI) utilizing electroencephalography (EEG). EEG is a non-invasive electrophysiological monitoring method used to record the brain’s electrical activity by measuring field potentials associated with cortical neural activity.

EEG can be used to control or communicate with a computer without using the natural neuromuscular pathways. A BCI recognizes the intent of the user through the processing of signals acquired with electrophysiological methods [19]. Past BCI research has primarily been focused on the communication aspect utilizing event-related potentials (e.g., P300 responses) [15, 18, 28, 41], steady-state visual evoked potentials (SSVEP) [27, 32, 35, 50] and sensorimotor rhythms (SMR) [38, 40, 48]. Furthermore, there is a great developing potential for BCIs to provide control of physical devices [9, 10, 17, 30, 31, 34, 44].

One of the key observations in EEG recordings is that the rhythmic neurophysiological activities recorded over the sensorimotor cortex are altered by movement, motor intention, or motor imagery (MI). The modulation manifests as amplitude decreases in the alpha or mu (8–13 Hz) and beta (14–26 Hz) frequency bands, also called event-related desynchronization (ERD). ERD is accompanied by an amplitude increase in the beta and gamma (30 Hz and higher) frequency band, also called event-related synchronization (ERS). Such rhythmic activity is referred to as SMRs [51]. Kobler et al. [29] has shown that directional information is also encoded around low-frequency delta band (0.2–5 Hz). From SMR-based BCI, motor intention or motor imagery can be recorded, which is the basis of neural control in such systems. Many studies have shown that people can learn to control the amplitude of SMR by using MI [14, 42, 47, 48].

In different experiments, participants were able to achieve both 2D and 3D control [14, 48]. Up to date, SMR BCIs offer the highest level of control in terms of degrees of freedom among all other signal components (e.g., evoked potentials or slow cortical potentials). The sources of SMR caused by movements or imagined movements of various body parts have been located in the primary sensorimotor cortex in a somatotopic manner [52]. In natural movement processes, the execution of movement and feedback (such as haptic information, proprioception, visual information, etc.) processes cannot be viewed as decoupled. Rather, movement actions are adjusted and refined during the execution based on sensory inputs that have a beneficial effect on the BCI control performance [20, 21]. One such sensory input is the vibrotactile guidance, which is used (in addition to visual guidance) in one of the two datasets analyzed in this paper. Besides correctly detecting the intent of the subject’s MI, one of the essential prerequisites of efficient active control of a BCI is the ability to detect when the user is not trying to issue any commands. This scenario is referred to as an Intentional Non-Control (INC) state [45]. It is very important to accurately detect when the user is trying to issue a command to minimize the possibility of false positive detection of control. In this paper, we aim to utilize information entropy to detect INC efficiently.

The concept of entropy was originally derived from thermodynamics as a measure of the disorder of a thermodynamic system. Its introduction to information theory has allowed quantification of the information content of a probability density function (PDF) [5, 6, 37]. Entropy-based signal complexity estimation of nonstationary signals in time–frequency plain can be interpreted as 2D energy distribution concentration [4, 6]. Time–frequency representations (TFRs) allow for straightforward interpretation and precise measurements of actual frequencies and the time instants at which they appear, as well as showing if the signal is monocomponent or multicomponent [6]. While for different applications, we can use different TFRs, in this paper, we focus on some TFRs that best describe our datasets and that are suitable for the calculation of the Rényi and Shannon entropy. TFRs are divided into Cohen’s class (quadratic or bilinear TFRs that are covariant by translation in time and frequency) and affine class (bilinear TFRs covariant by translation in time and dilation). Due to a high number of cross-terms present in the affine class TFRs [6], we focus on Cohen’s class TFRs. We tested several TFRs from the Cohen’s class and their reassigned counterparts. Reassigned TFRs utilize the reassignment method in order to improve signal sharpness and concentration. The reassignment method aims to move TFR values away from where they are computed towards the center of gravity, in order to produce better localization of the signal components [2]. For our work, we chose to utilize various TFRs interpreted as two dimensional PDFs and used them as an input for Rényi and Shannon entropy, effectively performing analysis of TFRs’ complexity and information content.

Recently, various studies have covered entropy applications in EEG SMR for different purposes. Spectral entropy of resting state (eyes closed) EEG was built and utilized as a biomarker to predict SMR BCI performance by Zhang et al. [53]. Tonin et al. [45] have shown that Shannon entropy can be utilized for the detection of INC state and thus improve the usability of a BCI by reducing unintentionally delivered commands during SMR BCI operations. They report an accuracy of 93.70% when predicting SMR detection. Another research focusing on utilizing entropy for motion detection (prediction) and INC state was done by Tortora et al. [46] where they reported an accuracy of 80% when detecting motion prediction. Jeong et al. [25] used dataset from Ofner et al. [39] and employed spectral filtering to improve movement-related cortical potentials detection. They achieved accuracy of 74% for detection of ‘elbow flexion’ movement. On the same dataset [23] achieved accuracy of 90.50% for detection of ‘hand open’ movement. Entropy for feature extraction was used in Chen et al. [12] where authors classified right and left-hand MI based on four combined entropy features (Shannon entropy based on amplitude, Shannon entropy based on phase, wavelet entropy, and sample entropy) and achieved average accuracies up to 85.71%. Sawant et al. [43] used a combination of empirical mode decomposition, common spatial patterns, power spectral entropy, and Walsh–Hadamard transform in order to acquire their features and achieve an average classification accuracy of 87.33% for right and left-hand MI. Ji et al. [26] utilized discrete wavelet transform, empirical mode decomposition, and approximate entropy to extract right and left-hand features which they classified with 85.71% accuracy.

In our work, we extract and compare amplitude features and entropy features (based on different TFRs) from the EEG SMR datasets where participants performed MI in congruence with visual guidance, visual and vibrotactile guidance, or visual cues only. After pre-processing and feature extraction, we performed classification and compared the features based on their classification accuracy and F1 score performance. The motivation for this research is to investigate the effectiveness of short-term entropy based on various TFRs for detecting MI in a more efficient manner (thus improving INC state detection), which could potentially improve the overall performance of BCI systems that use MI detection. Additionally, we aimed to investigate further the impact of vibrotactile guidance on MI detection, which could provide insights into potential improvements for BCI systems incorporating somatosensory feedback. Overall, the study aimed to contribute to the ongoing efforts to improve the performance and usability of BCI systems, particularly for individuals with motor disabilities who could benefit as potential end users of such MI-based BCI systems.

The paper is structured as follows. “Results and discussion” section provides results and their interpretation. In “Conclusion” section, we talk about conclusions and future work. In “Methods” section, the process of data acquisition, experiment setup, and processing of the data before the classification is described. Lastly, at the end of “Methods” section, we show the flowchart diagram of our proposed experiments and methods.

## Results and discussion

**Fig. 1:**
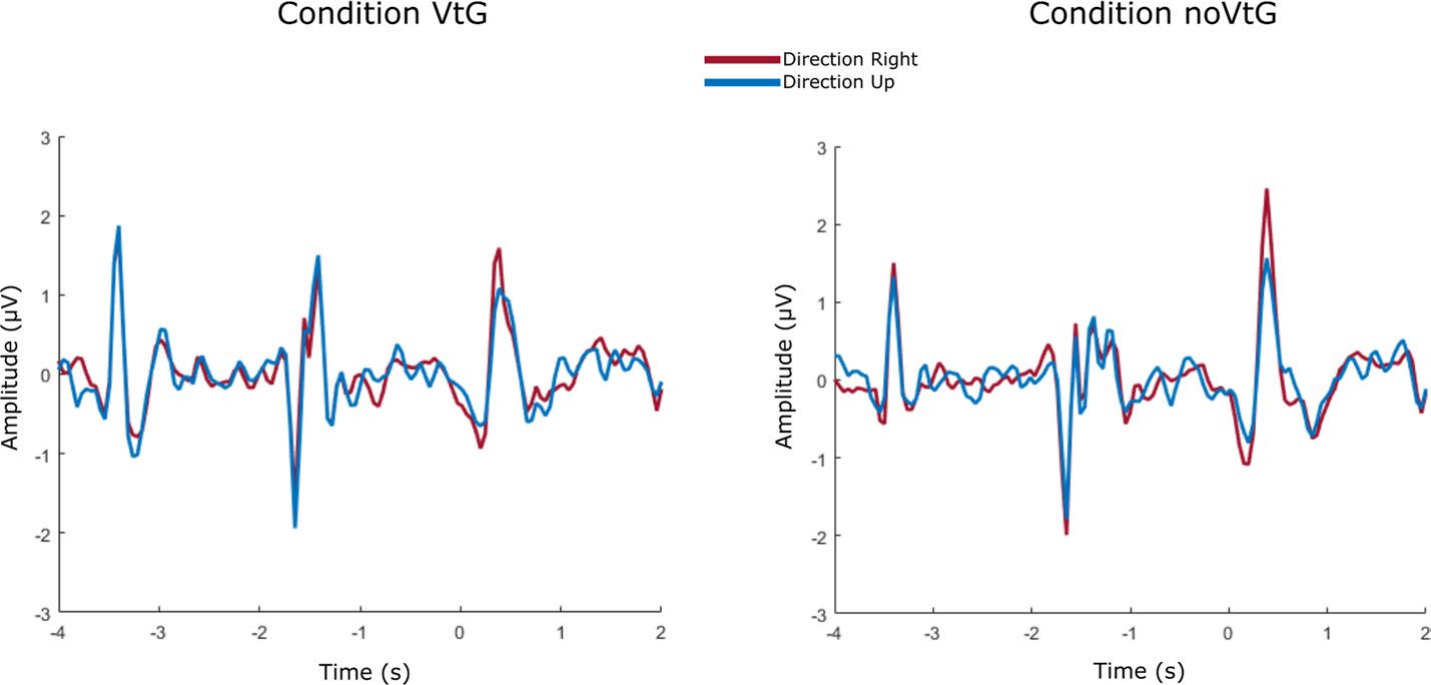
Grand average (across all participants) EEG amplitude potentials after the pre-processing described in “Pre-processing” section (Dataset 1), for each condition and each direction. Signals shown here are recorded on electrode location Cz

Results acquired after pre-processing of EEG amplitude features for Dataset 1, described in “Pre-processing” section, can be seen in Fig. 1. Results are shown for electrode location Cz, separately by each condition and direction. Here, we can observe slightly different visual evoked potentials (VEPs) in both conditions and both directions at certain time-points: appearance of the fixation cross ($t = -4$ s), appearance of the visual cue ($t = -2$ s) and start of the cue movement ($t = 0$ s). As we can observe, difference between directions in amplitude is not very prominent, yet it is present (notably in MI period).

**Fig. 2:**
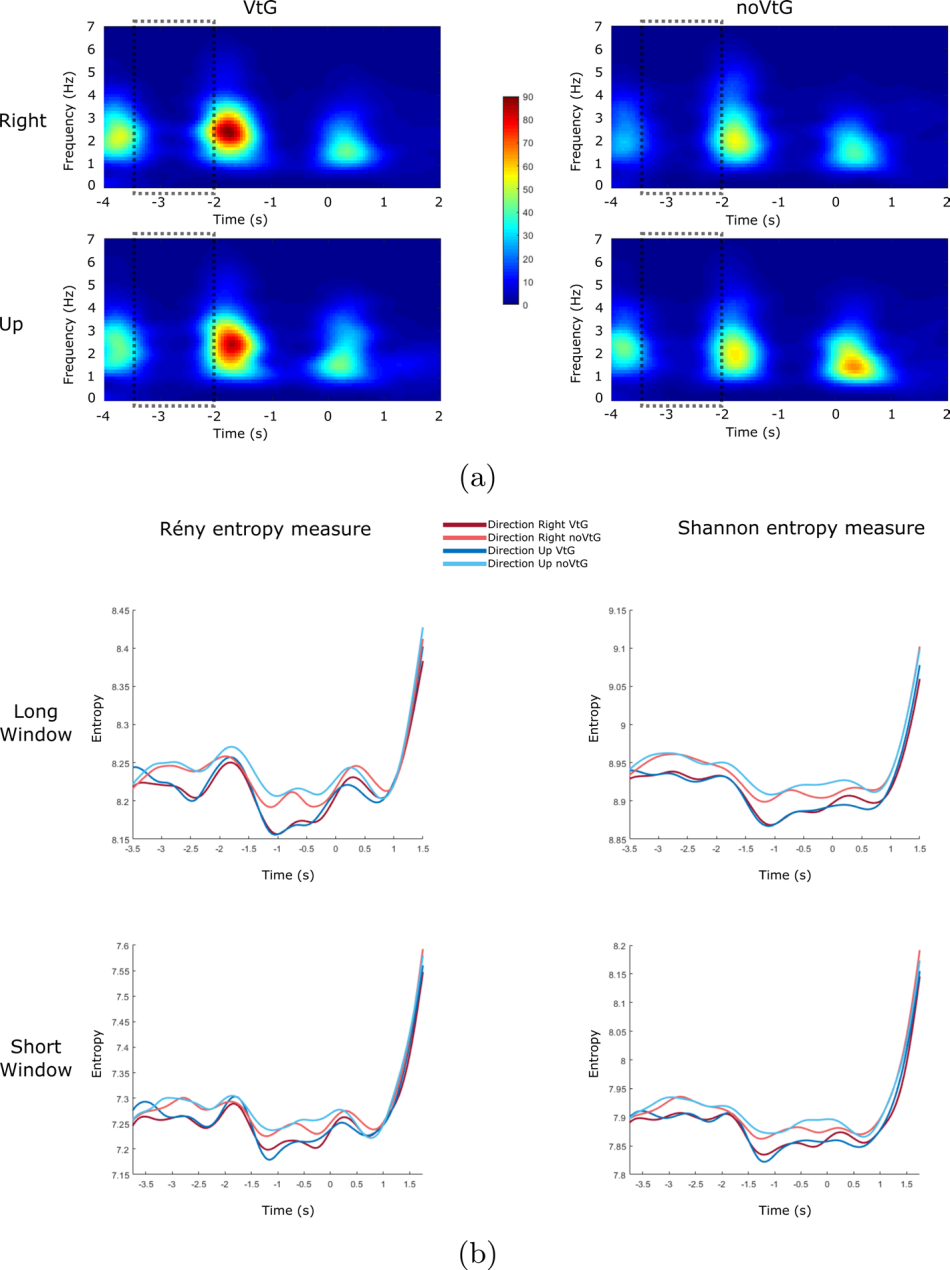
Spectrogram TFR, Rényi entropy and Shannon entropy example for each condition and each direction for amplitude features (Dataset 1), electrode location Cz. **a** Grand average (across all participants) Spectrogram representation TFR, baseline period is marked with dashed rectangles ($t = -3.5$ to $t = -2$). **b** Rényi entropy (left) and Shannon entropy (right) results for Spectrogram representation from **a**, for each window length (long window size is 1 s, and short window size is 0.5 s)

**Fig. 3:**
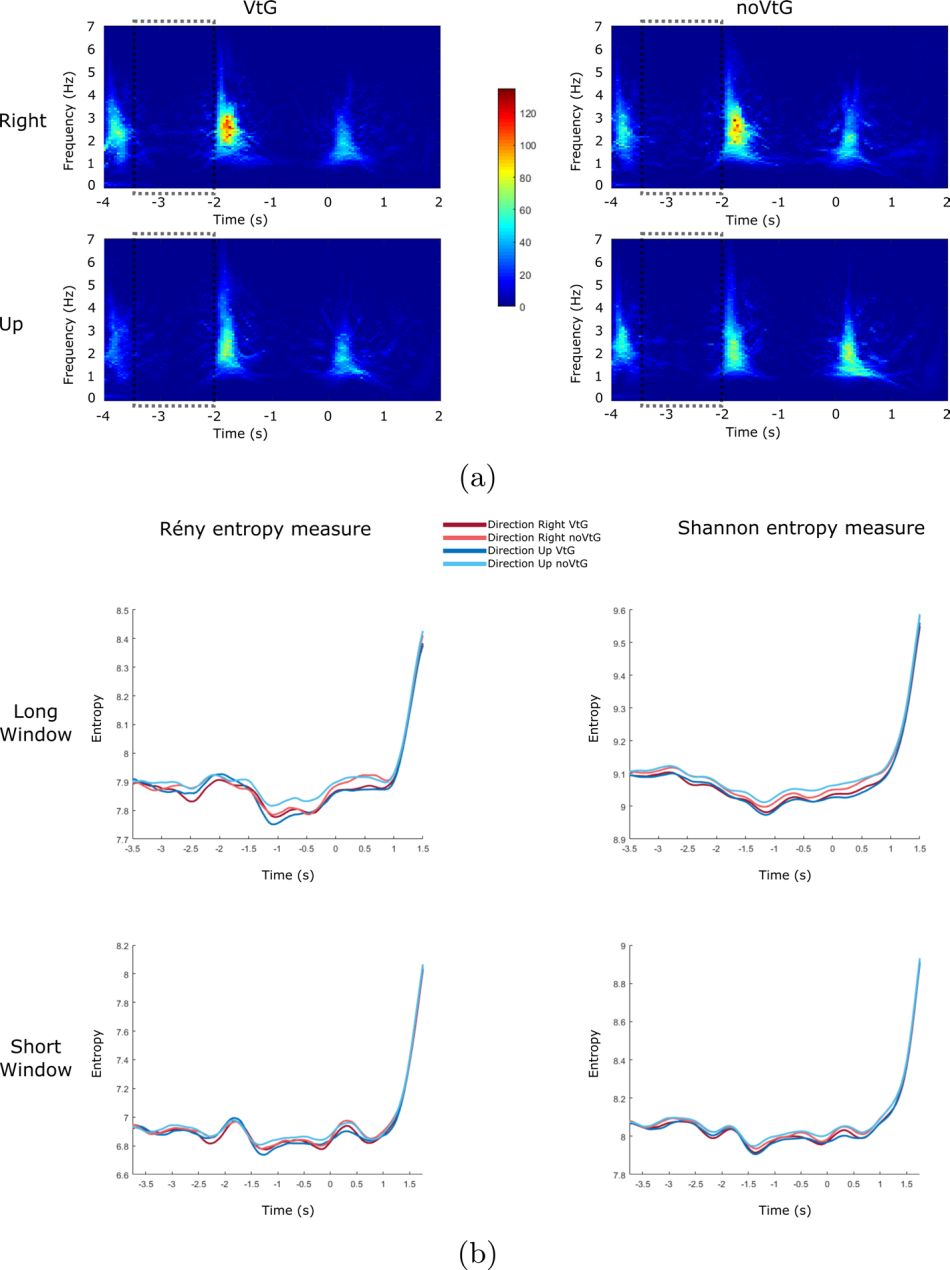
Reassigned Pseudo Wigner–Ville TFR, Rényi entropy and Shannon entropy example for each condition and each direction for amplitude features (Dataset 1), electrode location Cz. **a** Grand average (across all participants) Reassigned Pseudo Wigner–Ville TFR, baseline period is marked with dashed rectangles ($t = -3.5$ to $t = -2$). **b** Rényi entropy (left) and Shannon entropy (right) results for Reassigned Pseudo Wigner–Ville from **a**, for each window length (long window size is 1 s and short window size is 0.5 s)

Such amplitude features were used for calculation of different TFRs. Example of results for TFR, Rényi entropy and Shannon entropy can be seen in Figs. 2 and 3.

TFR used in Fig. 2 is a Spectrogram representation. In Fig. 2a we can see that condition VtG (visual guidance + vibrotactile guidance) has more prominent magnitude of Spectrogram during the entire trial for both directions, except for the ”Right” direction on noVtG (only visual guidance) around time $t = 0$.

Rényi and Shannon entropy results where TFR input to calculate the entropy was Spectrogram representation can be seen in Fig. 2b. Rényi and Shannon entropy give very similar results for both conditions and both directions at matching time-points, but as we can see, entropy varies during the trial.

For Rényi entropy, it is low before and during the baseline period ($t = -3.5$ to $t = -2$), increases during the beginning of pre-MI period ($t = -2$ to $t = -1.5$), decreases towards the end of pre-MI period ($t = -1.5$ to $t = 0$), and increases during MI period ($t = 0$ to $t = 1.5$). During the baseline period, Rényi entropy assumes lower values than during the MI period.

For Shannon entropy, values are lowest during the pre-MI period, and there is a less prominent increase during the pre-MI period (in comparison to baseline).

TFR used in Fig. 3 is Reassigned Pseudo Wigner–Ville. In Fig. 3a, we can see that, just like was the case with Spectrogram representation, condition VtG has stronger magnitude during key points throughout the trial for both directions.

Rényi and Shannon entropy results where TFR input to calculate the entropy was Reassigned Pseudo Wigner–Ville TFR can be seen in Fig. 3b. Resembling Spectrogram representation, Rényi and Shannon entropy give very similar results for both conditions and both directions at matching time-points, but entropy varies during the trial.

For Rényi entropy, it is low during baseline period ($t = -3.5$ to $t = -2$), increases temporary at the beginning of pre-MI ($t = -2$ to $t = -1.5$), decreases towards the end of pre-MI period ($t = -1.5$ to $t = 0$), and increases during MI period ($t = 0$ to $t = 1.5$). During the baseline period, Rényi entropy assumes lower values than during the MI period.

Similarly, for Shannon entropy, values are lowest during the pre-MI period and highest towards the end of the MI period, but with fewer variations during the entire trial.

Classification results for linear discriminant analysis with shrinkage regularization classifier (sLDA) in the form grand average (across all participants) accuracies/F1 during the MI period for amplitude features and Rényi entropy features with different TFRs and window sizes are shown in Tables 1 (long window Dataset 1), 2 (short window Dataset 1), 5 (long window Dataset 2) and 6 (short window Dataset 2). Results in the same form for Shannon entropy features with different TFRs and window sizes can be seen in Tables 3 (long window Dataset 1), 4 (short window Dataset 1), 7 (long window Dataset 2) and 8 (short window Dataset 2). TFRs used in said tables are: Spectrogram (tfrsp), Reassigned Spectrogram (tfrrsp), Gabor representation (tfrgabor), Reassigned Gabor spectrogram (tfrrgab), Pseudo Wigner–Ville (tfrpwv), Smoothed Pseudo Wigner–Ville (tfrspwv), Reassigned Pseudo Wigner–Ville (tfrrpwv), Reassigned Smoothed Pseudo Wigner–Ville (tfrrspwv).

**Table 1:** Dataset 1 long window ($w=1\,\text{s}$) and amplitude features grand average (across all participants) accuracy and F1 score of sLDA classification, shown by condition, types of features and types of TFRs calculated with Rényi entropy

	cond	Accuracy (%)								
TFR		–	tfrsp	tfrrsp	tfrgabor	tfrrgab	tfrpwv	tfrrpwv	tfrspwv	tfrrspwv
Feature type		Ampl.	Entropy	Entropy	Entropy	Entropy	Entropy	Entropy	Entropy	Entropy
Right vs up	VtG	**64.07**	52.87	54.83	53.62	53.96	53.61	53.67	52.74	52.56
	noVtG	**60.44**	52.72	53.33	52.56	54.21	52.38	51.10	51.49	52.64
Right vs base	VtG	86.91	64.68	60.78	69.97	66.95	75.04	**88.29**	66.92	64.52
	noVtG	85.68	64.85	61.48	67.42	68.47	76.25	**87.93**	67.84	63.43
Up vs base	VtG	85.81	60.48	59.90	67.20	67.29	71.94	**88.19**	63.55	60.93
	noVtG	84.59	65.05	61.91	67.72	69.57	77.77	**88.19**	68.30	64.40

		F1 score (%)								
Right vs up	VtG	**62.83**	51.08	54.41	53.35	52.95	52.15	54.20	51.28	51.57
	noVtG	**59.04**	51.96	52.69	52.28	53.62	50.92	49.78	50.88	51.94
Right vs base	VtG	84.19	64.44	60.35	69.87	66.54	72.15	**87.30**	66.50	64.23
	noVtG	82.52	63.60	61.31	67.34	67.55	73.35	**86.69**	67.21	63.86
Up vs base	VtG	82.30	59.76	59.49	67.50	66.47	68.59	**86.93**	63.17	60.47
	noVtG	80.55	64.14	61.84	67.87	68.50	75.03	**87.06**	67.40	63.84

The best results for a certain type of feature are shown in bold

**Table 2:** Dataset 1 short window ($w=0.5\,\text{s}$) features grand average (across all participants) accuracy and F1 score of sLDA classification, shown by condition, types of features and types of TFRs calculated with Rényi entropy

	cond	Accuracy (%)							
TFR		tfrsp	tfrrsp	tfrgabor	tfrrgab	tfrpwv	tfrrpwv	tfrspwv	tfrrspwv
Feature type		Entropy	Entropy	Entropy	Entropy	Entropy	Entropy	Entropy	Entropy
Right vs up	VtG	51.06	53.37	53.43	52.86	54.49	55.24	52.22	52.56
	noVtG	52.37	53.17	52.49	53.27	53.21	52.78	53.05	51.43
Right vs base	VtG	77.33	73.35	82.08	81.65	87.87	**98.39**	80.43	73.40
	noVtG	75.80	71.30	80.94	84.08	89.02	**98.78**	81.39	70.76
Up vs base	VtG	74.66	71.10	80.74	82.40	87.30	**98.44**	79.04	69.60
	noVtG	78.01	70.59	80.87	82.55	90.07	**98.65**	81.14	72.60

		F1 score (%)							
Right vs up	VtG	50.00	52.70	53.30	51.43	52.78	54.43	51.49	51.54
	noVtG	51.41	52.76	51.87	52.41	53.30	51.45	52.58	50.63
Right vs base	VtG	76.79	72.25	81.41	80.03	85.93	**98.29**	79.56	72.75
	noVtG	74.31	70.07	80.44	82.74	87.50	**98.71**	80.47	69.61
Up vs base	VtG	73.39	69.69	79.80	80.71	85.29	**98.33**	77.72	67.95
	noVtG	77.03	69.27	80.20	81.17	88.65	**98.55**	80.03	71.96

The best results for a certain type of feature are shown in bold

**Table 3:** Dataset 1 long window ($w=1\,\text{s}$) features grand average (across all participants) accuracy and F1 score of sLDA classification, shown by condition, types of features and types of TFRs calculated with Shannon entropy

	cond	Accuracy (%)							
TFR		tfrsp	tfrrsp	tfrgabor	tfrrgab	tfrpwv	tfrrpwv	tfrspwv	tfrrspwv
Feature type		Entropy	Entropy	Entropy	Entropy	Entropy	Entropy	Entropy	Entropy
Right vs up	VtG	53.69	52.73	53.14	51.51	52.83	52.74	52.41	53.01
	noVtG	53.38	52.78	52.42	53.52	52.71	53.19	52.88	51.99
Right vs base	VtG	65.22	59.51	79.40	58.68	83.24	**94.21**	71.41	62.65
	noVtG	65.40	59.29	77.82	58.38	85.32	**94.19**	71.52	63.41
Up vs base	VtG	62.54	60.97	77.30	58.04	81.64	**93.77**	68.39	61.69
	noVtG	67.60	58.41	79.25	57.83	86.23	**94.17**	72.41	61.32

		F1 score (%)							
Right vs up	VtG	52.51	51.84	52.72	50.07	52.19	52.45	51.16	52.00
	noVtG	53.16	52.92	52.05	52.82	51.78	51.85	53.23	51.42
Right vs base	VtG	64.29	58.48	78.91	57.51	81.30	**93.83**	70.98	61.84
	noVtG	63.91	58.07	77.22	57.80	83.54	**93.76**	71.01	62.81
Up vs base	VtG	62.03	59.24	76.76	57.00	79.44	**93.32**	67.83	61.04
	noVtG	66.73	57.43	78.73	57.26	84.87	**93.78**	71.81	60.76

The best results for a certain type of feature are shown in bold

**Table 4:** Dataset 1 short window ($w=0.5\,\text{s}$) features grand average (across all participants) accuracy and F1 score of sLDA classification, shown by condition, types of features and types of TFRs calculated with Shannon entropy

	cond	Accuracy (%)							
TFR		tfrsp	tfrrsp	tfrgabor	tfrrgab	tfrpwv	tfrrpwv	tfrspwv	tfrrspwv
Feature type		Entropy	Entropy	Entropy	Entropy	Entropy	Entropy	Entropy	Entropy
Right vs up	VtG	52.40	52.17	52.92	51.93	52.82	54.24	52.54	53.27
	noVtG	52.27	53.09	53.12	54.74	53.08	53.97	54.13	52.44
Right vs base	VtG	78.62	59.26	89.09	63.56	94.37	**99.69**	84.16	68.90
	noVtG	77.54	58.79	87.72	65.29	94.88	**99.87**	86.86	67.00
Up vs base	VtG	76.33	60.01	87.58	62.62	93.86	**99.58**	84.41	64.17
	noVtG	79.74	57.54	87.56	63.18	95.14	**99.75**	86.96	67.92

		F1 score (%)							
Right vs up	VtG	50.85	51.25	52.46	51.08	51.61	53.67	51.43	52.49
	noVtG	50.69	52.52	51.70	54.36	52.25	52.76	53.58	51.57
Right vs base	VtG	77.43	55.79	88.26	62.28	93.86	**99.67**	83.10	67.52
	noVtG	75.88	55.43	86.83	64.44	94.38	**99.87**	86.10	65.59
Up vs base	VtG	74.60	56.79	86.59	61.15	93.25	**99.56**	83.53	62.43
	noVtG	78.55	53.81	86.65	62.03	94.61	**99.75**	85.95	66.65

The best results for a certain type of feature are shown in bold

### Amplitude features classification results

Amplitude features (without entropy) accuracies/F1 can be seen in the first column of results in Table 1 (Dataset 1) and Table 5 (Dataset 2). As we can observe, the highest accuracy for amplitude features for directions right vs. up is achieved on Dataset 1 when using amplitude features with vibrotactile guidance (VtG), and it reaches a value of 64.07% which corroborates our previous findings [20] that VtG features perform slightly better than noVtG features (60.04% accuracy) when classifying different directions based on amplitude features. The highest overall accuracies for amplitude features is achieved on Dataset 1 when classifying MI (right or up) vs. baseline: between 84.59 and 86.91%.

**Table 5:** Dataset 2 long window ($w=1\,\text{s}$) and amplitude features grand average (across all participants) accuracy and F1 score of sLDA classification, shown by types of features and types of TFRs calculated with Rényi entropy

	Accuracy (%)								
TFR	–	tfrsp	tfrrsp	tfrgabor	tfrrgab	tfrpwv	tfrrpwv	tfrspwv	tfrrspwv
Feature type	Ampl.	Entropy	Entropy	Entropy	Entropy	Entropy	Entropy	Entropy	Entropy
EE vs EF	53.59	52.49	54.23	52.60	53.65	52.96	51.74	53.67	52.84
EE vs base	66.26	55.13	57.21	54.05	59.35	58.6	**71.28**	56.42	58.31
EF vs base	66.15	55.23	56.85	54.58	58.61	59.9	**70.95**	57.01	57.64

		F1 score (%)							
EE vs EF	52.72	52.42	53.90	52.27	53.08	52.46	50.99	53.04	52.41
EE vs base	55.85	54.68	56.01	52.8	58.56	56.34	**70.7**	54.7	56.1
EF vs base	56.18	54.61	55.91	53.93	58.27	57.32	**70.39**	55.16	56.14

The best results for a certain type of feature are shown in bold

Dataset 2 amplitude features when classifying different movements achieved accuracy of 53.59%, which is around chance level (55%). The highest Dataset 2 amplitude features accuracies are achieved when classifying MI—EE (elbow extension) or EF (elbow flexion)—vs. baseline: between 66.15 and 66.26% (shown in Table 5) which is similar to the findings in the study where this dataset originated (68% std 8%) [39].

The difference in the performance of our algorithm on Dataset 1 amplitude features and Dataset 2 amplitude features could be due to several reasons: paradigms are not the same. They are different in timings, movements that are imagined differ (movements right and up for Dataset 1 and elbow extension and flexion for Dataset 2) and paradigm of Dataset 1 contains vibrotactile guidance on certain trials, which kept the participants more engaged with the task. Another reason could be the positive effect of visual guidance (Dataset 1) in comparison to visual cue only (Dataset 2) [49]. One more reason for different performance could be the different electrode positions availability described in “Dataset 2” section.

### Rényi entropy classification results

For Dataset 1, the highest accuracy for long window Rényi entropy features is achieved with Reassigned Pseudo Wigner–Ville representation TFR and is equal to 88.29% for VtG right vs. base (shown in Table 1), which is a slight increase of 1.31% compared to amplitude VtG features right vs. base (86.91%).

The highest accuracy for short window Rényi entropy features for Dataset 1 and the highest Rényi entropy accuracy overall is achieved for noVtG features for direction right vs. base when calculating with Reassigned Pseudo Wigner–Ville TFR and is equal to 98.78% (shown in Table 2), which is an increase of 13.10% when compared to amplitude noVtG features right vs. base (85.68%).

For Dataset 2, the highest accuracy for long window Rényi entropy features is also achieved with Reassigned Pseudo Wigner–Ville representation TFR and is equal to 71.28 for EE vs. base% (shown in Table 5), which is an increase of 5.02% in comparison to amplitude features EE vs. base (66.26%).

The highest accuracy for short window Rényi entropy features For Dataset 2 and the highest Rényi entropy accuracy overall for Dataset 2 is achieved for movement EF vs. base when calculating with Reassigned Pseudo Wigner–Ville TFR and is equal to 87.17% (shown in Table 6), which is an increase of 21.02% when compared to amplitude features EF vs. base (66.15%).

**Table 6:** Dataset 2 short window ($w=0.5\,\text{s}$) features grand average (across all participants) accuracy and F1 score of sLDA classification, shown by types of features and types of TFRs calculated with Rényi entropy

	Accuracy (%)							
TFR	tfrsp	tfrrsp	tfrgabor	tfrrgab	tfrpwv	tfrrpwv	tfrspwv	tfrrspwv
Feature type	Entropy	Entropy	Entropy	Entropy	Entropy	Entropy	Entropy	Entropy
EE vs EF	53.37	55.97	52.51	54.61	51.42	53.85	52.91	53.19
EE vs base	63.13	67.76	63	76.25	72.39	**87.01**	66.88	58.23
EF vs base	64.2	68.68	62.55	76.43	72.24	**87.17**	65.87	58.37

		F1 score (%)						
EE vs EF	51.81	55.42	51.78	54.83	51.74	53.31	52.18	52.33
EE vs base	61.16	65.72	61.16	74.48	69.6	**85.67**	65.85	53.65
EF vs base	62.41	66.87	60.45	74.29	69.7	**85.73**	64.47	53.23

The best results for a certain type of feature are shown in bold

### Shannon entropy classification results

The accuracies/F1 for both long window and short window Shannon entropy features is also best in the Reassigned Pseudo Wigner–Ville TFR (shown in bold in Tables 3, 4, 7 and 8) as it was the case with Rényi entropy, for both datasets.

**Table 7:** Dataset 2 long window ($w=1\,\text{s}$) features grand average (across all participants) accuracy and F1 score of sLDA classification, shown by types of features and types of TFRs calculated with Shannon entropy

	Accuracy (%)							
TFR	tfrsp	tfrrsp	tfrgabor	tfrrgab	tfrpwv	tfrrpwv	tfrspwv	tfrrspwv
Feature type	entropy	entropy	entropy	entropy	entropy	entropy	entropy	entropy
EE vs EF	53.14	52.9	53.36	53.69	52.34	52.26	51.86	51.7
EE vs base	55.55	57.64	56.26	59.17	63.67	**76.86**	60.28	62.1
EF vs base	55.9	58.69	57.36	58.36	63.58	**76.84**	59.41	61.87

		F1 score (%)						
EE vs EF	53.13	53.13	53.12	53.48	52.77	51.91	51.84	51.77
EE vs base	54.95	56.24	55.69	57.5	61.91	**76.11**	58.57	60.73
EF vs base	55.44	56.82	57.29	56.52	61.66	**76.02**	57.66	60.41

The best results for a certain type of feature are shown in bold

**Table 8:** Dataset 2 short window ($w=0.5\,\text{s}$) features grand average (across all participants) accuracy and F1 score of sLDA classification, shown by types of features and types of TFRs calculated with Shannon entropy

	Accuracy (%)							
TFR	tfrsp	tfrrsp	tfrgabor	tfrrgab	tfrpwv	tfrrpwv	tfrspwv	tfrrspwv
Feature type	Entropy	Entropy	Entropy	Entropy	Entropy	Entropy	Entropy	Entropy
EE vs EF	52.84	53.09	52.65	52.85	52.15	53.05	51.69	51.79
EE vs base	63.36	59.92	70.53	62.92	84.45	**94.82**	70.01	61.77
EF vs base	62.66	60.2	69.49	63.62	84.3	**95.27**	69.02	61.82

		F1 score (%)						
EE vs EF	51.76	52.38	51.79	52.73	53.25	53.12	51.86	52.13
EE vs base	62.2	56.69	69.67	61.34	83.39	**94.61**	69.04	58.03
EF vs base	60.9	57.02	68.69	61.45	83.11	**95.04**	68.23	58.28

The best results for a certain type of feature are shown in bold

For Dataset 1, the highest accuracy for long window Shannon entropy features is equal to 94.21% and is achieved for VtG right vs. base when calculating with Reassigned Pseudo Wigner–Ville TFR (shown in Table 3). This is an increase of 7.30% when compared to amplitude VtG features right vs. base (86.91%) and an increase of 5.92% when compared to long window Rényi entropy Reassigned Pseudo Wigner–Ville representation amplitude VtG features right vs. base (88.29%).

The highest accuracy for short window Shannon entropy features for Dataset 1 and the highest overall accuracy is achieved for noVtG features for direction right vs. base when calculating with Reassigned Pseudo Wigner–Ville TFR (shown in Table 4) and is equal to 99.87%, which is an increase of 14.19% when compared to amplitude noVtG features right vs. base (85.68%).

For Dataset 2, the highest accuracy for long window Shannon entropy features is equal to 76.86% and is achieved for EE vs. base when calculating with Reassigned Pseudo Wigner–Ville TFR (shown in Table 7 which is an increase of 10.60% in comparison to amplitude features EE vs. base (66.26%)

The highest accuracy for short window Shannon entropy features For Dataset 2 and the highest overall accuracy for Dataset 2 is achieved for movement EF vs. base when calculating with Reassigned Pseudo Wigner–Ville TFR and is equal to 95.27% (shown in Table 8), which is an increase of 29.12% when compared to amplitude features EF vs. base (66.15%). This result is also, to our bes knowledge, better than previous work on the same dataset, including [23, 25, 39].

Compared to Rényi entropy, Shannon entropy measure has lower increase during the baseline period relative to increase/variation during the MI period (seen in Figs. 2 and 3), which explains the higher accuracies/F1 that we achieved with Shannon entropy in comparison to Rényi entropy.

As can be observed in Tables 1, 2, 3, 4, 5, 6, 7, and 8, some of our Rényi and Shannon entropy features performed very well (up to 99.87%) in our main goal of MI detection (MI vs. baseline), neither of them performed very well in detection of different directions or movements (right vs. up or EE vs. EF) which indicates that the TFRs’ complexity and information content contained in different directions of a same limb could not be detected in this way, which goes in line with the similarities between directions shown in Figs. 2 and 3 explained in “Results and discussion” section.

## Conclusion

The brain–computer interfaces based on sensorimotor rhythms are a point of interest to many researchers globally. With advances in sensors, signal processing algorithms, and intelligent control solutions, better accuracy of the systems is achieved every day.

This paper proposes a new method for processing and detection of MI data and provides a comparison of amplitude features, Rényi and Shannon short-term entropy features (with various window sizes) used for classification of signals when MI task was guided by visual guidance, visual and vibrotactile guidance or visual cue only. Methods were tested and developed on Dataset 1 from our previous study [20] and additionally tested on publicly available and commonly used Dataset 2 from [39] study. Amplitude features give better classification accuracy results than entropy features for classification of different directions or movements (up to average 64.07%, Dataset 1), but entropy features give better classification accuracy results than amplitude features for classification of MI vs. baseline (resulting in average accuracy up to 99.87% for short window Shannon entropy for Dataset 1 and average accuracy up to 95.27% for Dataset 2). When considering different TFRs as input to entropy measure, the best results were acquired when using the Reassigned Pseudo Wigner–Ville. Our findings have shown that the proposed approach can increase average accuracy up to 14.19% when using the proposed entropy features instead of amplitude features in cases of classifying MI against the baseline period for Dataset 1 and can increase average accuracy up to 29.12% in the same situation for Dataset 2.

From our analysis, we can conclude that MI detection (i.e., classification of MI vs. baseline) is very efficient when entropy is used on certain types of TFRs with our proposed processing and notably on paradigm with vibrotactile guidance (Dataset 1). The approach of processing and classification described in our paper can be utilized for efficient detection of MI which is important in real case scenario of usage of BCI where unwanted movement detection should not occur, and movement detection should be triggered only when there is an actual MI. Furthermore, we can conclude that vibrotactile guidance has neither positive nor negative impact on accuracy of MI detection, however, we corroborated previous findings that the congruent vibrotactile guidance used in MI experiment has a slight positive impact on accuracy of detecting different directions or movements (when used on amplitude features).

As for our future work, we plan to expand our dataset with data augmentation methods and try to improve classification accuracy with some state-of-the-art classification methods from the machine learning field. Besides this, we plan on recording a different MI experiment where movement imagination of various limbs would be used in order to assess the impact of usage of various TFRs on short-term entropy in such paradigm.

## Methods

In this paper, we used two datasets to develop and test our methods. Dataset 1 is acquired from “Feel Your Reach” project from [20, 36]. We chose to use this particular dataset because of its variety (two visually guided classes—direction ‘Up’ and direction ‘Right’; and two conditions—MI with vibrotactile guidance and MI without vibrotactile guidance) and simplicity (simple linear continuous center-out MI). Dataset 1 is the dataset that our methods were developed on and for that reason in this paper we will be focusing mostly on this dataset to describe and introduce our methods. Dataset 2 is the SMR dataset acquired from BNCI Horizon 2020 project [39]. This dataset was chosen in addition to Dataset 1 to test our methods on one of the commonly used SMR datasets that are available online. Although Dataset 2 uses visual cue instead of visual guidance and it does not use two different conditions (i.e., it does not have vibrotactile guidance), it has simple MI tasks and paradigm very similar to our originally used Dataset 1.

### Dataset 1

**Fig. 4:**
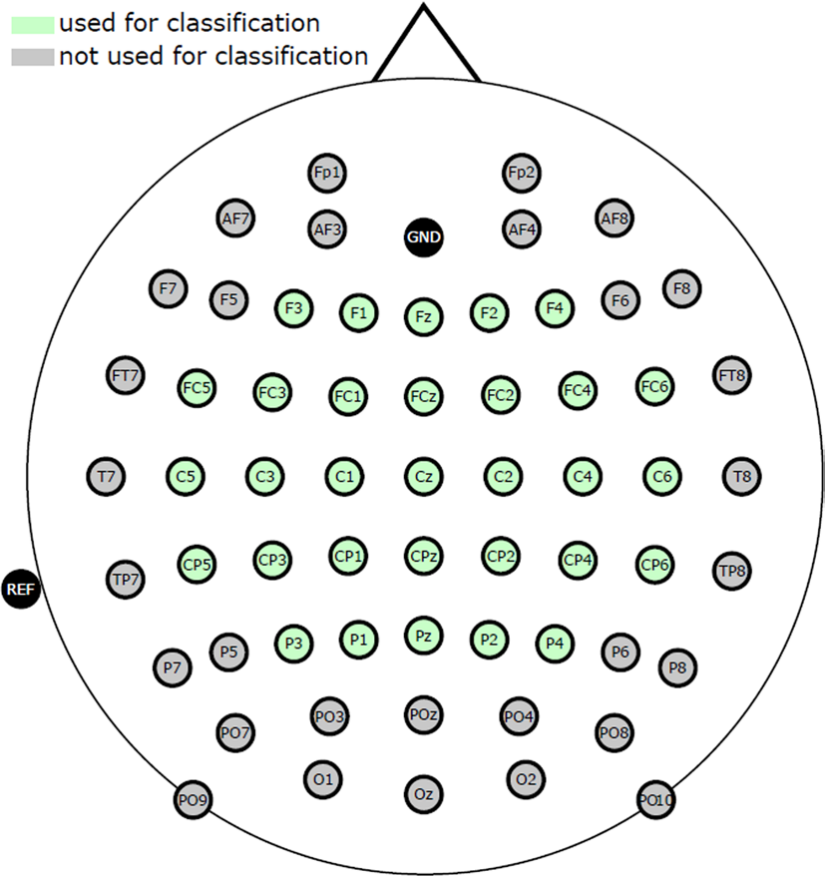
International 10/20 EEG system cap montage [20]

EEG and electrooculogram (EOG) were recorded from 61 and 3 actiCap electrodes, respectively, using two BrainAmp amplifiers (Brain Products GmbH, Gilching, Germany) at a sampling rate of 1 kHz. Electrodes were arranged according to the international 10/20 EEG system, shown in Fig. 4, where 61 channels were used for EEG, and 3 channels were used for EOG. Later, in the processing and classification of data, only 31 channels around the motor-related section were used (marked in green on Fig. 4).

**Fig. 5:**
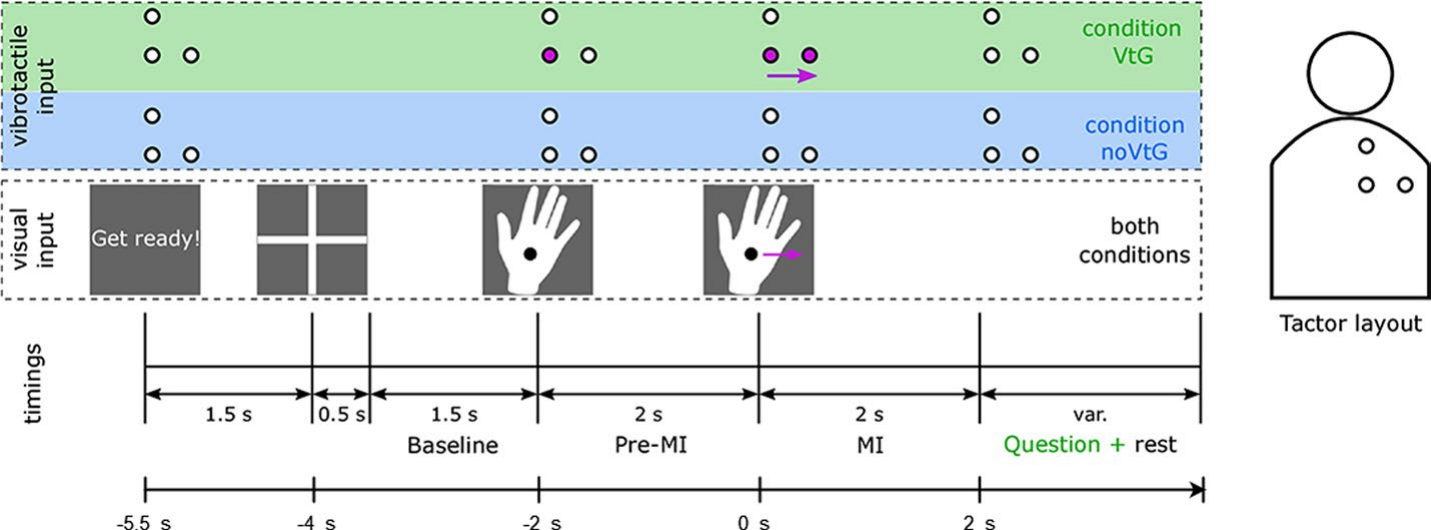
Paradigm of the experiment trial for Dataset 1 [20]. Top row of the image (shaded in green and blue) depicts the position and activation of the tactors that delivered vibrotactile input (guidance) in congruence with the visual input (depicted in the middle row of the image). Timings are shown in the bottom row of the image

Data were recorded from 15 participants. Participants were between 21 and 32 years old (avg 25.36, std 3.4); 7 males and 8 females. All participants were right-handed. Out of the 15 participants, 10 of them had prior experience with MI. Each participant participated in one session where six runs were recorded. Each run consisted of either VtG (visual guidance + vibrotactile guidance) or noVtG (only visual guidance) MI tasks (3 VtG and 3 noVtG runs). In condition VtG, vibrotactile guidance was delivered by three tactile actuators (C-2 tactors—Engineering Acoustics Inc., Casselberry, USA) which were attached to the inside of an elastic shirt to stimulate the right shoulder blade [20]. Those tactors delivered haptic vibrotactile guidance in form of a moving sensation on participant’s shoulder. In each run, there are 40 trials. Each trial was 7.5 s long, and MI happened during a 2-s period, as shown in Fig. 5. The participants were visually informed to “Get ready!” at the beginning of each trial, 1.5 s before the appearance of the fixation cross. The fixation cross was displayed for 2 s, the latter 1.5 s of which were later utilized as a baseline period (used for processing and classification). During this period, participants were instructed to fixate their gaze on the fixation cross and relax. The monitor then displayed the visual cue, a right hand with a fixation point. During the 2-s pre-MI interval, it remained stationary before moving either to the right or up at a consistent speed. Participants were instructed to perform the MI in accordance with the movement of the cue and fixate their gaze on a fixation point (black dot in the middle of the hand cue). In condition VtG, participants were subsequently asked to determine if the vibrotactile guidance was congruent to the visual guidance in this trial and to answer with a (keyboard) keypress [20].

### Dataset 2

EEG and EOG were recorded from 61 and 3 active electrodes, respectively, using four g.tec amplifiers (g.tec medical engineering GmbH, Austria) at a sampling rate of 512 Hz. 8th order Chebyshev bandpass filter was used to filter between 0.01 and 200 Hz. Electrodes were arranged similarly to the montage shown in Fig. 4, only the 31 electrodes marked in green were in matching positions to our Dataset 1 montage. Because of this differences in montage, for Dataset 2 we used only electrodes marked in green and EOG electrodes for pre-processing, processing and classification.

**Fig. 6:**
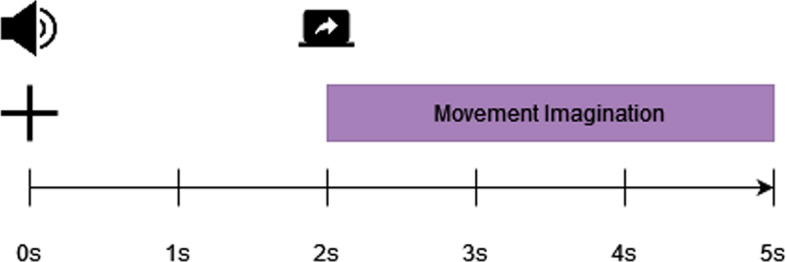
Paradigm of the experiment trial for Dataset 2 [39]

Data were recorded from 15 participants. Participants were between 22 and 40 years old (mean 27, std 5); 6 males and 9 females. All participants except one were right-handed. Each participant participated in one MI session where ten runs were recorded. Each run consisted of different MI tasks where stationary visual cues were presented on screen in front of a participant. Tasks were: elbow flexion, elbow extension, supination, pronation, hand close, hand open. In each run, there are 36 trials (6 of each 6 tasks). Each trial was 5 s long, and MI happened during a 3-s period, as shown in Fig. 6. The fixation cross was displayed for 2 s, the latter 1.5 s of which were later utilized as a baseline period (used for processing and classification). During this period, participants were instructed to fixate their gaze on the fixation cross. The monitor then displayed the stationary visual cue indicating the required task (one of six movements). Participants were instructed to perform the MI in accordance with the given stationary visual cue [39]. In our work, we used only two tasks: EF (elbow flexion) and EE (elbow extension). These tasks were selected because of their similarity to tasks from Dataset 1 (MI ‘up’ and ‘right’).

Some of the differences in Dataset 2 from Dataset 1 are: paradigms are different in timings, movements that are imagined differ (movements right and up for Dataset 1 and elbow extension and flexion for Dataset 2) and paradigm of Dataset 1 contains vibrotactile guidance on certain trials. Dataset 1 also contains visual guidance while Dataset 2 contains stationary visual cue only.

### Pre-processing

Before classification, data were pre-processed in the following manner:

1. Data were downsampled to 200 Hz, bandpass filtered between 1 and 40 Hz (4th order zero-phase Butterworth filter [8]), epoched to a relevant period (Dataset 1: from $t=-5.5$ s to $t=2$ s as shown in Fig. 5. Dataset 2: from $t=0$ s to $t=5$ s as shown in Fig. 6).
2. Bad trials were rejected based on amplitude threshold and artifact presence, using EEGLAB MATLAB toolbox [13]
3. Independent Component Analysis (ICA) [13, 33] was performed for each participant separately. For Dataset 1 it was performed on 61 EEG channels (giving 61 independent components). The remaining 3 EOG channels were used for artifact removal. For Dataset 2 it was performed on 31 EEG channels (giving 31 independent components). The remaining 3 EOG channels were used for artifact removal. For both datasets, only relevant independent components (IC) were kept—with SASICA [11] and manual IC rejection.
4. Data were further filtered in bands of interest with the 4th order zero-phase Butterworth filter, specifically 0.2 to 5 Hz for amplitude features. This band was selected due to best performance of amplitude features shown in our previous study [20].
5. Amplitude features were then further downsampled to 20 Hz.
6. 31 relevant channels around the motor-related section were selected for further analysis and processing (marked in green on Fig. 4).

From here, classification was done based on the amplitude features. For the classification of the entropy features, we first calculated different TFRs from amplitude features, then, the short-term entropy (Rényi or Shannon) were calculated from those TFRs with two different window sizes, i.e., long window ($w=1$ s) and short window ($w=0.5$ s), in order to inspect the influence of various window lengths on entropy results. Window size of $w=0.5$ s was selected as a lower limit because the further decrease of window size has a negligible increase of accuracy as an effect but at the cost of an increase in computational time. In this work, we performed a classification comparison of amplitude features, Rényi entropy features, and Shannon entropy features. Both Rényi and Shannon entropy were calculated from different TFRs.

### Entropy calculation

The entropy is used as an indicator of the energy distribution concentration of the TFR [37]. The interpretation is that a highly concentrated TFR with a small number of components has lower entropy than a signal with a large number of signal components [7]. Short term entropy was calculated on the moving window of either long window ($w = 1$ s) or short window ($w = 0.5$ s) width and 50 ms step, over each trial, for each channel separately. Value at a certain time-point is calculated from a window that reaches on both sides of that time-point equidistantly.

The Rényi entropy is calculated as:

1 $$R^\alpha _x={\frac{1}{1-\alpha }}\log _2 {\left ( \int _{-\infty }^\infty \int _{-\infty }^\infty {\text{TFR}}_x^{\alpha }(t,f) \text{d}t \text{d}f \right )}, $$

where $R^\alpha _x$ is the $\alpha$ order Rényi entropy for the $\text{TFR}_x$ (due to oscillation reducing effects, we have set Rényi entropy order to $\alpha = 3$ as in Baraniuk et al. [5]). Since certain TFRs can assume negative values due to the interferences, we have taken absolute values of the calculated TFR before calculating an entropy.

To compare with the Rényi entropy, we also utilized the Shannon entropy defined as:

2 $$I_{x}=-\int _{-\infty }^{+\infty } \int _{-\infty }^{+\infty } \text{TFR}_x(t,f) \log _{2} \text{TFR}_x(t,f) \text{d}t \text{d}f. $$

### Time–frequency representations

Time–frequency representations may be divided into two classes: Cohen’s class and Affine class. Cohen’s class TFRs are quadratic or bilinear TFRs that are covariant by translation in time and frequency. Affine class TFRs are bilinear TFRs covariant by translation in time and dilation. In our experiment, we focus on Cohen’s class TFRs because they yielded better results in our preliminary studies, most likely due to a high number of cross-terms present in the affine class TFRs. Four different TFRs and their four reassigned counterparts were used as part of this analysis.

The reassignment method is used in order to improve signal sharpness or concentration. The reassignment method aims to move TFRs values away from where they are computed towards the center of gravity, in order to produce better localization of the signal components [2]. The key principle of the method is that values of a certain distribution have no reason to be symmetrically distributed around a certain time–frequency point where they are usually calculated, but rather at the center of gravity of this domain, which gives a better representation of the local energy distribution of the signal [3].

For the entropy calculation, we used the following Cohen’s class TFRs:

1. Spectrogram, which is a simple Cohen’s class TFR and can be interpreted as bilinear energy distribution. Spectrogram has a trade-off between the time resolution and frequency resolution as a drawback and good interference deduction if two signal components are sufficiently far apart [3, 22]. The spectrogram is calculated as:
  3 $$S_{x}(t, \nu )=\left| \int _{-\infty }^{+\infty } x(u) h^{*}(u-t) e^{-j 2 \pi \nu u} \text{d}u\right| ^{2},$$
  where *h* is a frequency smoothing window. We can interpret the spectrogram as a measure of the energy of the signal contained in the time–frequency domain centered on the point $(t, \nu )$ [3].
2. Reassigned Spectrogram, introduced as an attempt to improve the spectrogram’s localization to produce sharper representation of signal components [3]. The reassigned spectrograms is calculated with the equation
  4 $$\begin{aligned}  \text{RS}_{x}\left( t^{\prime }, \nu ^{\prime }; h\right) & =\int \int _{-\infty }^{+\infty } S_{x}(t, \nu ; h) \delta \left( t^{\prime }-\hat{t}(x; t, \nu )\right) \\ & \quad \delta \left( \nu ^{\prime }-\hat{\nu }(x; t, \nu )\right) \text{d}t \text{d} \nu , \end{aligned} $$
  where $\delta$ is reassignment operation, $(t^{\prime }, \nu ^{\prime })$ is the value of reassigned spectrogram and $(\hat{t}, \hat{v})$ is a center of gravity of the signal energy distribution around (*t*, *v*). The reassigned spectrogram also uses the phase information of the short-time Fourier transform, and not only its squared modulus as it is the case with the spectrogram [3].
3. Gabor representation was introduced to remove the highly oscillated cross-terms without significantly altering desirable properties, i.e., it can balance the resolution and cross-term interference [6, 16]. The Gabor representation is calculated as:
  5 $$G_{x}[n, m; h]=\sum _{k} x[k] h^{*}[k-n] \exp [-j 2 \pi m k], $$
  where $G_{x}[n, m; h]$ are Gabor coefficients (*n*, *m*).
4. Reassigned Gabor spectrogram which is a reassigned spectrogram utilizing Gabor representation [16]. Calculated with Eq. (4), but utilizing Gaussian window instead of frequency smoothing window, thus allowing faster computation [3].
5. Pseudo Wigner–Ville distribution is based on Wigner–Ville distribution (WVD) which has many desirable properties such as preservation of time and frequency shifts and energy conservation. Since WVD has a drawback of producing strong cross-terms in multicomponent signals [6], Pseudo Wigner–Ville distribution introduces windowing operation which is equivalent to frequency smoothing of WVD [3, 24]. As a result, cross-terms are attenuated comparing to regular WVD. It is calculated as:
  6 $$\text{PW}_{x}(t, \nu )=\int _{-\infty }^{+\infty } h(\tau ) x(t+\tau / 2) x^{*}(t-\tau / 2) e^{-j 2 \pi \nu \tau } \text{d} \tau , $$
  where *h* is frequency smoothing operation.
6. Smoothed Pseudo Wigner–Ville is a pseudo Wigner–Ville distribution that utilizes time and frequency smoothing (in contrast to frequency only smoothing that is present in Pseudo Wigner–Ville) in order to smooth the signal in time and frequency domain [1]. The previous compromise of spectrogram between time and frequency resolutions is now replaced by a compromise between the joint time–frequency resolution and the level of cross-terms (more smoothing results in poorer resolution) [3]. It is defined as:
  7 $$ \begin{aligned} \text{SPW}_{x}(t, \nu ) & =\int _{-\infty }^{+\infty } h(\tau ) \int _{-\infty }^{+\infty } g(s-t) x(s+\tau / 2) \\ & \quad x^{*}(s-\tau / 2) \text{d}s\; e^{-j 2 \pi \nu \tau } \text{d} \tau , \end{aligned}$$
  where *g* is time smoothing operation.
7. Reassigned Pseudo Wigner–Ville is a Pseudo Wigner–Ville TFR that utilizes reassignment method [3], and is calculated as:
  8 $$ \begin{aligned} \text{RPWV}_{x}\left( t^{\prime }, \nu ^{\prime }; h\right) & =\iint _{-\infty }^{+\infty } P W V_{x}(t, \nu ; h) \\ & \quad \delta \left( t^{\prime }-\hat{t}(x; t, \nu )\right) \delta \left( \nu ^{\prime }-\hat{\nu }(x; t, \nu )\right) \text{d}t \text{d} \nu . \end{aligned} $$
8. Reassigned Smoothed Pseudo Wigner–Ville is a Pseudo Wigner–Ville TFR that utilizes reassignment method and separable (time and frequency) smoothing function. It is defined as:
  9 $$\begin{aligned} \text{RSPWV}_{x}\left( t^{\prime }, \nu ^{\prime }; g, h\right) & =\iint _{-\infty }^{+\infty } \text{SPWV}_{x}(t, \nu ; g, h) \\ & \quad \delta \left( t^{\prime }-\hat{t}(x; t, \nu )\right) \delta \left( \nu ^{\prime }-\hat{\nu }(x; t, \nu )\right) \text{d}t \text{d}\nu ,  \end{aligned}$$
  where *g* is the time smoothing window.

### Classification

We performed classification of several feature types:

- Amplitude features
- Entropy features (long window, i.e., $w=1$ s)
- Entropy features (short window, i.e., $w=0.5$ s).

Every one of the feature types was classified based upon several class distributions.

For Dataset 1:

- Condition VtG, directions: right vs up
- Condition noVtG, directions: right vs up
- Condition VtG: direction right vs baseline
- Condition VtG: direction up vs baseline
- Condition noVtG: direction right vs baseline
- Condition noVtG: direction up vs baseline.

For Dataset 2:

- Movements: EE vs EF
- Movement EE vs baseline
- Movement EF vs baseline.

For the classification of selected features, we used linear discriminant analysis with shrinkage regularization (sLDA) classifier and did the classification in a fivefold way wherein each fold 75% of the dataset was used for training and cross-validation, and 25% for testing. Accuracies and F1 scores are calculated in the following manner: first, average accuracy/F1 for every single participant separately was calculated; second, a grand average accuracy/F1 across all participants was calculated (average accuracy/F1 of all participants); third, grand average accuracy/F1 during MI period was taken into consideration as an end result of classification accuracy/F1.

The accuracy is calculated as:

10 $$ \text{acc}= \frac{\text{TP} + \text{TN}}{n}, $$

and F1 is calculated as:

11 $$ \text{F}1= \frac{\text{TP}}{\text{TP} + 0.5\;(\text{FP}+\text{FN})}, $$

where *n* is the number of trials, TP is the number of True Positives, TN is the number of true negatives, FP is the number of False Positives, and FN is the number of False Negatives. F1 score represents the harmonic mean of the precision and recall of the classification.

**Fig. 7:**
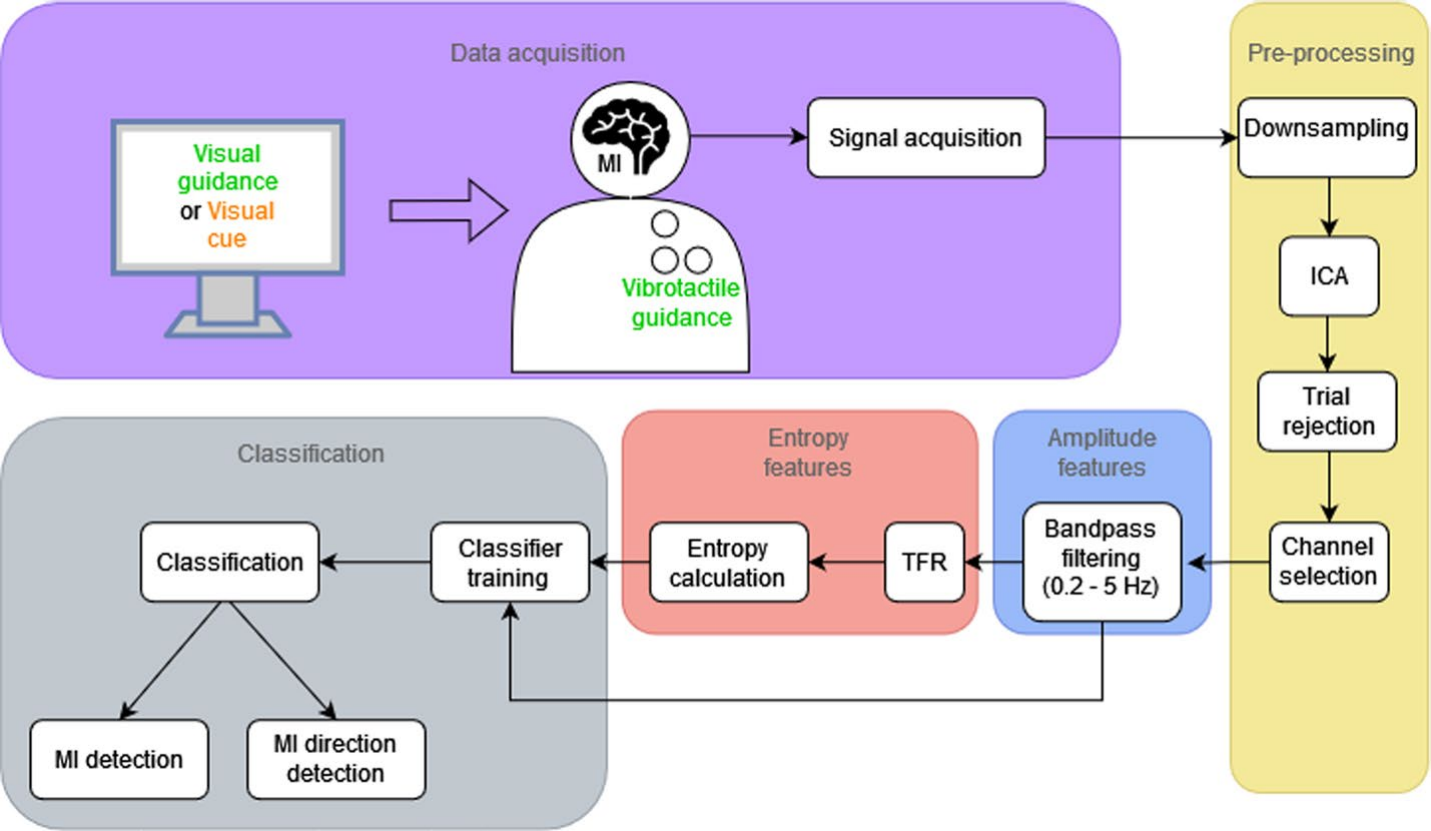
Flowchart diagram of the data acquisition (purple), processing pipeline (yellow for pre-processing, blue for amplitude features processing, and red for entropy features processing), and classification (gray) phases of our proposed method. Note that the Dataset 1 uses Visual guidance and a combination of Visual guidance and Vibrotactile guidance, and the Dataset 2 uses Visual cue only

To summarize “Methods” section, in Fig. 7 we can see a flowchart diagram of our proposed method, including data acquisition, processing, and classification phases.

## Data Availability

Data and the programming code used as part of this research can be obtained from authors on a request.
